# Feasibility and diagnostic accuracy of paramedic-performed prehospital point-of-care ultrasound: a retrospective observational study

**DOI:** 10.1186/s13049-026-01595-4

**Published:** 2026-03-28

**Authors:** Adrian Boehm, Tobias Bexten, Michael Stanley, Dieter Westphal, Robert Buder, Ferdinand Konrad-Borgstaedt, Peter Benoehr

**Affiliations:** 1 Philipps-Universität Marburg, Marburg, Germany; 2 Helios Dr. Horst Schmidt Clinic Wiesbaden, Clinic for Interdisciplinary Intensive Medicine and Intermediate Care, Prehospital POCUS Project, German Red Cross Fulda, Wiesbaden, Germany; 3 Helios Dr Horst Schmidt Clinic Wiesbaden, Clinic for Interdisciplinary Intensive Medicine and Intermediate Care, Wiesbaden, Germany; 4 German Professional Association of Paramedics (DBRD), Prehospital POCUS Project, German Red Cross Fulda, Lübeck, Germany; 5 Medical School Brandenburg, Prehospital POCUS Project, German Red Cross Fulda, Neuruppin, Germany; 6 Clinic for Internal Medicine, Nephrology and Medical Intensive Care, Prehospital POCUS Project, German Red Cross Fulda, Charité – Universitätsmedizin, Berlin, Germany; 7 Asklepios Klinik Nord - Heidberg, Clinic for Emergency Medicine, Prehospital POCUS Project, German Red Cross Fulda, Hamburg, Germany; 8 Clinic for Internal Medicine, Nephrology, Klinikum Fulda gAG, Universitätsmedizin Marburg Campus Fulda, Marburg, Germany

**Keywords:** Point-of-care ultrasound, POCUS, Paramedic, Prehospital care, Diagnostic accuracy, Feasibility, Emergency medical services

## Abstract

**Background:**

Point-of-care ultrasound (POCUS) is increasingly being used in prehospital emergency medicine. While physician-performed prehospital ultrasound is well established, evidence regarding the feasibility and diagnostic accuracy of paramedic-performed POCUS in real-world settings remains limited.

**Methods:**

We conducted a retrospective observational cohort study evaluating paramedic-performed POCUS following a structured, multimodal training program. Twenty-one certified paramedics performed handheld ultrasound examinations in prehospital emergencies using standardized protocols with an observation period of 24 months, starting from March 2023. Feasibility, utilization patterns, diagnostic accuracy, and perceived clinical impact were assessed using standardized documentation. Hospital diagnoses served as the reference standard, based on radiological, sonographic, and/or clinical documentation.

**Results:**

A total of 169 ultrasound examinations were performed on 144 patients. The overall diagnostic performance achieved a sensitivity of 87.9% and specificity of 92.7%. Diagnostic accuracy, defined as the concordance between prehospital POCUS-based working diagnoses and final in-hospital diagnoses, was particularly strong for lung ultrasound (pneumothorax, pulmonary edema, pneumonia and pleural effusion; sensitivity 91.7%, specificity 100%) and eFAST (sensitivity 100%, specificity 96.5%), while for the abdominal ultrasound examinations, the specificity was 70% and sensitivity was 71.43%. Ultrasound findings influenced logistical and clinical decision-making in a substantial proportion of missions, including changes in transport urgency (36.1%) and hospital destination (18.1%), whereas emergency room prealerts were avoided in 9.7% of cases. Training resulted in significantly more positive attitudes regarding feasibility, clinical relevance, and image of paramedic-performed ultrasound. Concerns regarding time delay and workload were markedly reduced.

**Conclusions:**

Paramedic-performed prehospital POCUS is feasible after structured training and can be integrated into routine prehospital care. Prospective studies should further assess the diagnostic accuracy, reliability, and clinical impact of paramedic-performed POCUS in the prehospital setting.

**Supplementary Information:**

The online version contains supplementary material available at 10.1186/s13049-026-01595-4.

## Introduction

Rapid diagnostic assessment is essential in prehospital emergency medicine, particularly for patients presenting with trauma, dyspnea, or shock. Therefore, point-of-care ultrasound (POCUS) has become an integral diagnostic tool in emergency departments and physician-staffed emergency medical services (EMS), supporting early diagnosis and therapeutic decision-making [1–3].

Technological advances have enabled the deployment of compact, handheld ultrasound devices suitable for prehospital environments. Multiple studies have demonstrated the feasibility and diagnostic utility of physician-performed prehospital ultrasound [4]. In contrast, evidence for paramedic-performed POCUS remains limited, particularly regarding independent application, diagnostic accuracy, and real-world feasibility.

Internationally, EMS systems differ substantially in staffing models and scope of practice. It can be broadly distinguished between the Anglo-American model, where paramedics are the primary prehospital providers, and the Franco-German model, where the EMS is usually physician-led [5]. However, many countries now blend elements of both philosophies to match local culture, resources, geography, and politics [6, 7]. European helicopter EMS (HEMS) shows wide variation: dual medical crew vs. single provider, paramedic‑ vs. physician‑crewed, with staffing perceived to affect safety [8]. Expanding paramedic diagnostic capabilities may therefore enhance early clinical assessment.

Previous studies on paramedic-performed POCUS have largely focused on educational outcomes or on simulated environments [9, 10]. Data evaluating real-world application and diagnostic agreement with in-hospital findings remain scarce.

Therefore, we address this gap by evaluating paramedic-performed prehospital POCUS after structured training, focusing specifically on feasibility, utilization patterns, and diagnostic accuracy. The objectives of this study were as follows: A) To assess the feasibility of independent prehospital POCUS performed by paramedics after structured training. B) To analyze real-world utilization patterns of predefined ultrasound protocols. C) To evaluate diagnostic accuracy by comparing prehospital ultrasound findings with final in-hospital diagnoses based on X-ray, CT-Scan, sonographic, and/or clinical documentation. D) To assess the perceived impact of ultrasound findings on prehospital decision-making, and E) General attitudes self-perceived effect before and after structured POCUS training.

## Methods

### Study design

This study was conducted as a retrospective observational cohort assessing the feasibility and diagnostic accuracy of paramedic-performed prehospital POCUS following structured training and conducted in accordance with the STROBE guidelines.

The study was performed within a regional German emergency medical service operating a two-tiered EMS system. The implementation of POCUS was conducted as a structured educational and clinical integration project. Twenty-one certified paramedics were included. All participants voluntarily completed the POCUS training program.

The POCUS curriculum included a blended learning module, face-to-face lectures, hands-on workshops, simulation-based case training, and 2 days of supervised clinical practice in an emergency department. Details are given in Table 1.

**Table 1:** Blended Learning curriculum

Training Step	Core Activities	Learning Focus
1 – Blended Learning (Self-study)	• Study POCUS fundamentals via textbook • Curated video libraries and FOAM resources • Pretests or knowledge checks optional	• anatomy knowledge • ultrasound physics • pathology recognition
2 – Interactive Lectures	• Media-enhanced PowerPoint® sessions • Live demonstration of sonographic findings • Continuous Q&A and engagement elements	• Conceptual understanding • mental models • diagnostic pathways
3 – Skills Workshops	• Hands-on image acquisition on phantoms and peers • Probe orientation, depth, gain, M-mode, Doppler where applicable • Vascular access training: in-plane & out-of-plane	• Psychomotor skills, • handling confidence, • artifact recognition
4 – Case-Based Simulation	• Scenario training using xABCDE structure • Sonography only displayed when correct probe position achieved • Structured debriefing after each case	• Clinical reasoning • protocol adherence • team communication
5 – Clinical Placement (Supervised ED shifts)	• Real patient scanning under physician supervision • Application of lung, eFAST, cardiac POCUS, RUSH & vascular access in emergencies • Feedback on image quality and decision integration	• Transfer to real-world • workflow integration, • autonomy

### Ultrasound equipment

Participants were equipped with a handheld ultrasound device (Butterfly iQ + ®) for independent prehospital use.

### Protocols

The training focused on standardized protocols: 1) lung ultrasound (LUS), 2) extended focused assessment with sonography for trauma (eFAST), 3) focused cardiac ultrasound, 4) rapid ultrasound in shock and hypotension (RUSH), and 5) ultrasound-guided peripheral venous access [11–15] (Tables 2 and 3). All participants were trained according to these standardized protocols; however, individualized scanning (e.g., gallbladder, hydronephrosis) was permitted and left to the participant’s decision. Here, the abdominal scans included assessment for free intra-abdominal fluid, gallbladder pathology (e.g., cholelithiasis), and bowel pathology suggestive of ileus. Leg vein ultrasound refers to a focused venous compression ultrasound (CUS) performed to assess for deep vein thrombosis (DVT). Urological ultrasound examinations were performed as focused KUB-type scans (kidneys and bladder), primarily to assess for hydronephrosis and urinary retention.

**Table 2:** List of Ultrasound Protocols Implemented in the Training Program

Protocol	Clinical purpose (Rephrased Description)
Lung ultrasound (LUS)	• Rapid bedside assessment for pneumothorax, pulmonary edema, pneumonia and pleural effusion
Extended Focused Assessment with Sonography for Trauma (eFAST)	• Evaluation for free fluid in the peritoneal and pleural spaces including pericardial effusion and detection of pneumothorax in trauma patients to support immediate triage decisions
Focused Cardiac Ultrasound (FoCUS)	• Point-of-care evaluation of cardiac function, volume status, and pericardial effusion to differentiate between shock states and detect reversible causes of hemodynamic compromise
Rapid Ultrasound in Shock and Hypotension (RUSH)	• Systematic scanning protocol integrating heart, lungs, and abdominal vessels to determine the underlying cause of undifferentiated shock
Ultrasound-guided peripheral intravenous access (USGIV)	• Use of real-time ultrasound guidance to obtain peripheral venous access in patients with difficult intravenous access, improving speed and success rates
Echocardiography in Advanced Life Support (ELS)	• Cardiac ultrasound during resuscitation to distinguish between true cardiac standstill and potentially reversible pathology (e.g., tamponade), guiding advanced life support decisions

**Table 3:** Self-perceived influence of ultrasound on clinical decision-making before and after structured POCUS training

Clinical indication	Before training median (IQR)	After training median (IQR)	*p* value
Breathing problems (B-problems)	3 (3–4)	4 (4–5)	<.001
Circulation problems (C-problems)	3 (3–4)	4 (4–5)	0.004
Ultrasound-guided vascular access	3 (3–4)	4 (4–5)	0.024
Echocardiography in advanced life support (ALS)	4 (3–5)	4 (4–5)	0.326
Polytrauma scenarios	4 (4–5)	4 (4–5)	0.608

Values represent median ratings with interquartile ranges (IQRs) on a 5-point Likert scale (1 = strongly disagree, 5 = strongly agree). Pre- and posttraining comparisons were performed using the Wilcoxon signed-rank test

### Data collection

#### Self-perception and training effect

Before and after the training, all 21 participants were asked to fill out a questionnaire that focused on self-perceived learning effect and attitude toward the use of ultrasound in the prehospital setting. The perceived impact on clinical decision-making was assessed by paramedic self-report and reflects subjective appraisal.

#### Real-world data

The observation period was 24 months, starting from March 2023. After the training, the participating paramedics took the POCUS device out on every ambulance mission and used it as needed. Following each ultrasound application, paramedics completed a standardized questionnaire documenting 1) indication for ultrasound, 2) protocol applied, 3) key ultrasound findings, and 4) subjective assessment of impact on prehospital management. Details of the questionnaire are given in the Appendix.

Final diagnoses were established by retrospective review of hospital discharge summaries and discharge letters, including all documented diagnoses, diagnostic reports, and/or clinical findings.

Based on these objectives, we defined the endpoints as follows:

Primary endpoint: The primary endpoint was feasibility, defined as the ability of trained EMS personnel to independently indicate POCUS, select an appropriate standardized protocol, and perform a complete and interpretable examination without real-time supervision, as documented in routine prehospital records. As feasibility cannot be captured by a single measure, it was assessed multidimensionally by (A) diagnostic accuracy, evaluated by agreement between prehospital ultrasound findings and final hospital diagnoses, and (B) utilization frequency of ultrasound protocols. Utilization-based feasibility was estimated using aggregate mission and staffing data.

Secondary endpoints: (1) perceived impact on prehospital decision-making; (2) general attitudes toward ultrasound before and after training; and (3) self-perceived effects of ultrasound diagnostics before and after structured POCUS training.

### Statistical analysis

Diagnostic accuracy was assessed using contingency tables, and sensitivity and specificity were calculated where applicable. The effect size is given as Cramers V and OR. Ordinal questionnaire data were analyzed using the Wilcoxon test. No formal sample size calculation was performed due to the exploratory nature of the study. Data were analyzed descriptively using *RStudio® (Version 2025.09.02* + *418).*

### Ethics approval and consent to participate

This study was conducted as a retrospective observational analysis of fully anonymized data. According to local regulations and German law, formal ethics approval was not needed. Due to the retrospective nature of the study and the exclusive use of anonymized data, informed consent from individual participants was not required and was waived by the Ethics Committee of the State Medical Association of Hesse, Germany (Ethik-Kommission bei der Landesärztekammer Hessen; reference number: V/1/sja/nic 2024-3811-AF).

## Results

### Primary endpoint

#### Feasibility of independent prehospital POCUS use

A total of 169 prehospital ultrasound examinations were performed on 144 patients, including successful ultrasound-guided peripheral i.v. catheter placements.

#### Utilization frequency of ultrasound protocols

**Fig. 1:**
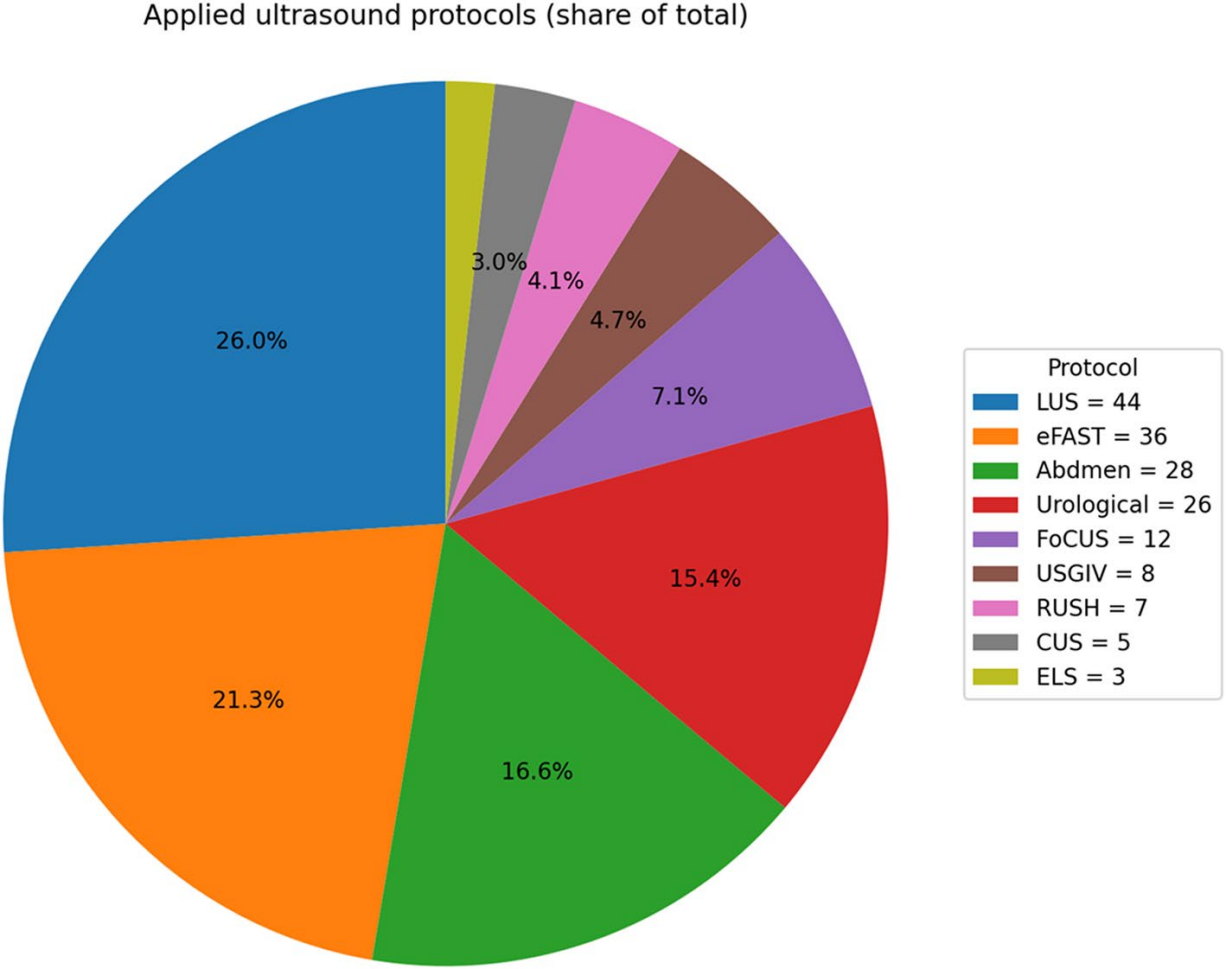
Distribution of applied prehospital ultrasound protocols. Lung ultrasound (LUS), Extended Focused Assessment with Sonography for Trauma (eFAST), Focused Cardiac Ultrasound (FoCUS), Rapid Ultrasound in Shock and Hypotension (RUSH), ultrasound-guided peripheral intravenous access (USGIV), and echocardiography in advanced life support (ELS). Values are shown as percentages of all performed examinations and absolute numbers

During the study period, paramedics conducted 169 POCUS examinations using a variety of emergency ultrasound protocols. The most commonly applied modalities were focused or individualized examinations (42%), followed by lung ultrasound (26%) and eFAST (21%). Advanced protocols such as RUSH (4%) and ultrasound-guided i.v. access (5%) were used selectively, while echocardiography in advanced life support (ELS) accounted for only 2% of total scans. A total of 54 individualized examinations were performed, including 28 abdominal and 26 urological examinations. Details are given in Fig. 1.

Of the 169 prehospital ultrasound examinations, 101 cases could be matched with final hospital diagnostic results and were included in the diagnostic accuracy analysis.

Overall, paramedic-performed POCUS demonstrated a sensitivity of 87.9% (true positives: 29; false negatives: 4) and a specificity of 92.7% (true negatives: 63; false positives: 5).

Diagnostic performance was particularly strong for the primary emergency protocols. For eFAST, documented as positive or negative, the sensitivity was 100% (3/3), and the specificity was 96.6% (28/29). For lung ultrasound, the sensitivity was 91.7% (11/12), and the specificity was 100% (13/13). The detected pathologies included six B-line patterns, four pleural effusions, and one consolidation/pneumonia.

A total of 17 abdominal ultrasound examinations were included in the analysis. Seven patients had relevant abdominal pathologies, of whom five were correctly identified prehospitally (sensitivity 71.4%), including one ruptured spleen, one ruptured kidney, one abscess of the iliac crest, and two cases of gallbladder stones. Two cases were missed, including one perforation of a hollow organ and one patient with gallbladder stones. Among 10 patients without abdominal pathology, seven were correctly classified (specificity 70%), with three false-positive findings, including two prehospital suspicions of cholecystitis and one case of free fluid.

Sixteen individualized urological ultrasound examinations were included, with a sensitivity of 90% (9/10) and a specificity of 83.3% (5/6). The observed pathologies included five cases of urinary retention, one case of hydronephrosis, and two cases of bladder tamponade. One case of hydronephrosis was falsely positive, and one case of urinary retention was falsely negative.

The remaining examinations were not included in sensitivity or specificity calculations because of low case numbers and the absence of pathological findings. These included one femoral vein ultrasound, one ELS examination, four RUSH examinations, and five FoCUS examinations.

Further details are provided in Fig. 3.

**Fig. 2:**
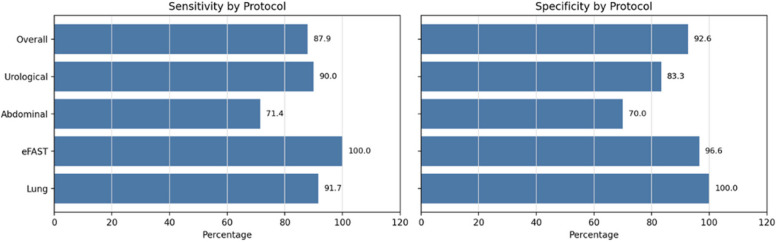
Sensitivity and specificity of paramedic-performed prehospital ultrasound by protocol. Bars represent protocol-specific diagnostic accuracy compared with final in-hospital diagnoses

Figure 2: Diagnostic accuracy by Protocol.

**Fig. 3:**
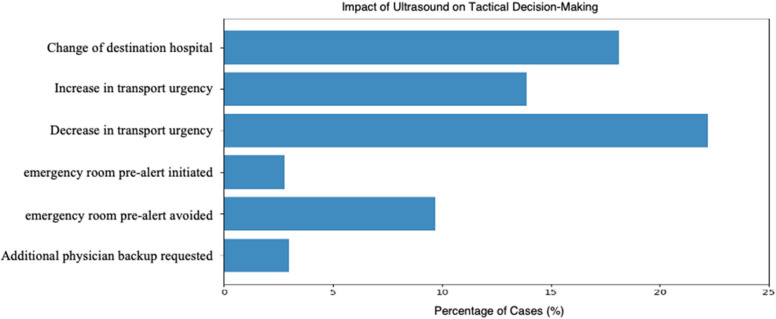
Impact of ultrasound on tactical decision-making in prehospital care. Bars represent the percentage by which the POCUS influenced the tactical decision

Perceived Impact on Prehospital Clinical Decision-Making: Based on sonographic findings, transport urgency was modified in 36.1% of cases. In 22.2% of cases, it was possible to decrease the transport urgency; in 13.9% of cases, it was increased. The destination hospital was changed in 18.1% of cases. Notably, POCUS enabled the avoidance of unnecessary emergency room prealerts in 9.7% of missions, in accordance with national, indication-specific German guidelines [16, 17], yet in 2.8% POCUS led to an initiation. In 44.4% of cases, prehospital ultrasound confirmed the suspected diagnosis. Self-perceived diagnostic clarity improved in 52.8% of cases by excluding one or more differential diagnoses. Sonographic findings led to medication administration in 4.9%. Unintended delays related to ultrasound were rare (< 1%), with most prolonged on-scene times judged as clinically appropriate due to the diagnostic benefit (31.2%). The duration was not affected in 54.2% of cases. Details are given in Fig. 3.

### Secondary endpoints

#### Self-perception and training effect

Following the training, all 21 participants completed a questionnaire that focused on self-perceived learning effect and attitude toward the use of ultrasound in the prehospital setting. Here, the participants rated the influence of ultrasound on hospital destination decision-making significantly higher than before. Regarding specific clinical indications, the median rating increased from 3 (IQR 3–4) before training to 4 (IQR 4–5) after training (*p* < 0.001) for breathing problems. Similarly, for circulation problems, the median increased from 3 (IQR 3–4) to 4 (IQR 4–5) (*p* = 0.004).

For ultrasound-guided vascular access, the perceived usefulness also increased significantly from a median of 3 (IQR 3–4) before training to 4 (IQR 4–5) after training (*p* = 0.024).

No significant changes were observed for echocardiography in advanced life support (median 4 [IQR 3–5] vs. 4 [IQR 4–5]; *p* = 0.326) or polytrauma scenarios (median 4 [IQR 4–5] both before and after training; *p* = 0.608). Details are given in Table 1.

Finally, following the training, participants reported a significant reduction in perceived barriers to prehospital ultrasound use. The perceived difficulty of learning ultrasound decreased from a median of 4 (IQR 3–4) before training to 2 (IQR 2–3) after training (*p* < 0.001).

Agreement with the statements that ultrasound diagnostics should remain reserved for physicians decreased significantly from a median of 2 (IQR 1–3) to 1 (IQR 1–2) (*p* = 0.013) and that ultrasound diagnostics should be physician-only decreased from 2 (IQR 1–3) to 1 (IQR 1–2) (*p* = 0.007).

**Fig. 4:**
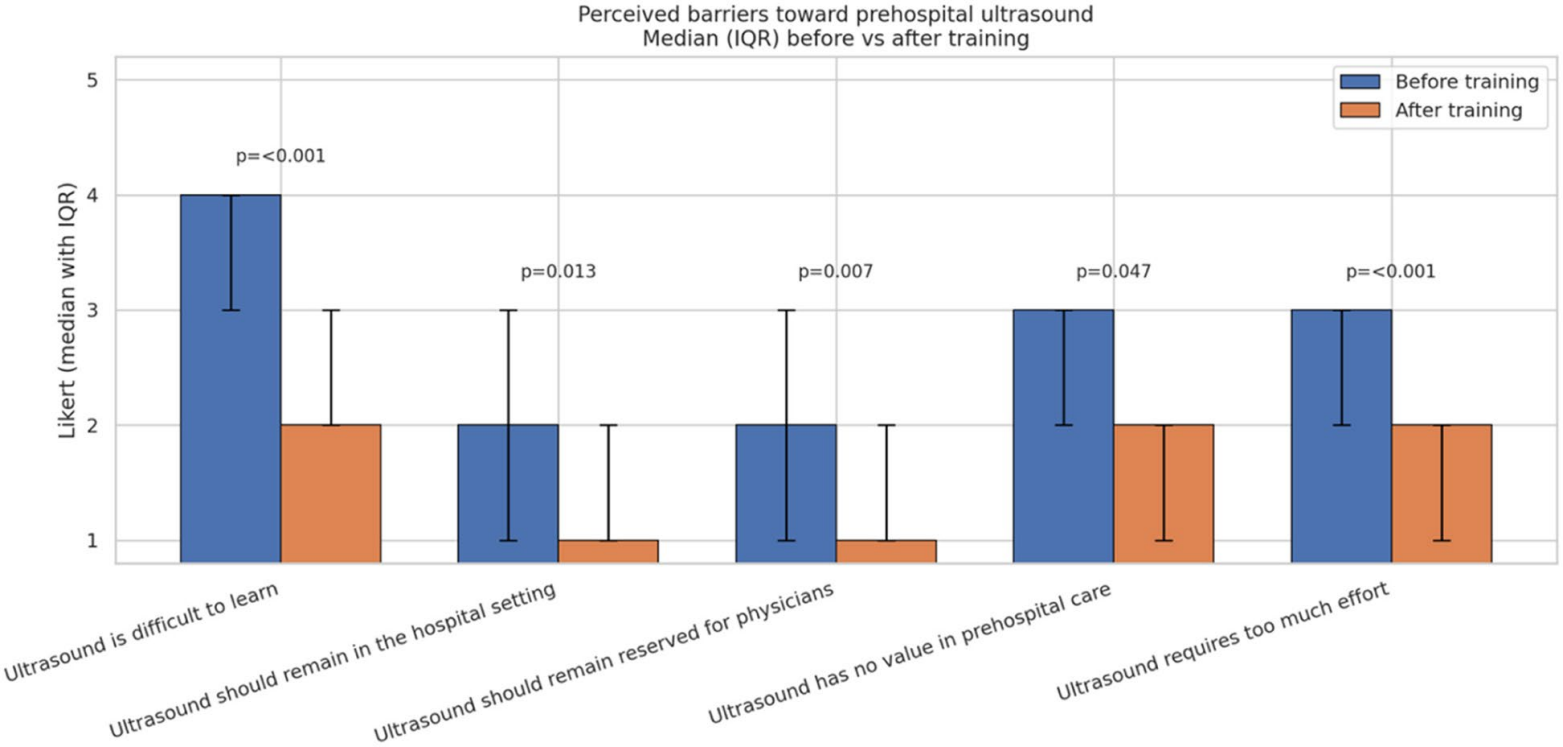
General attitudes toward ultrasound diagnostics before and after structured POCUS training. Data represent agreement on a 5-point Likert scale (1 = strongly disagree, 5 = strongly agree). All statements show a significant shift toward more positive attitudes

Furthermore, the perceived lack of relevance of ultrasound in the prehospital setting declined from a median of 3 (IQR 2–3) to 2 (IQR 1–2) (*p* = 0.047). Perceived effort associated with ultrasound diagnostics also decreased significantly from a median of 3 (IQR 2–3) before training to 2 (IQR 1–2) after training (*p* < 0.001) (Fig. 4).

## Discussion

This study demonstrates that structured ultrasound training for paramedics is feasible and associated with significant self-reported gains in knowledge, confidence, and perceived clinical relevance. Participants showed improvement not only in advanced protocols such as RUSH and ultrasound-guided vascular access but also in established applications such as eFAST, indicating both an initial knowledge gap and the effectiveness of the training program. The results suggest that focused education can meaningfully enhance understanding of point-of-care ultrasound (POCUS) in the prehospital setting. This aligns with previous observations [1]. Here, recently, Weber et al. reported significant gains in knowledge of the fundamentals of POCUS and image interpretation after providing an 8 h eFAST course [9]. Other investigators addressed the operator-dependent nature of POCUS and the lack of structured training as significant barriers. Therefore, they introduced an extensive training program that not only relies on hands-on learning and in-person instruction but also incorporates AI-driven feedback systems as well as AI-assisted probe manipulation and self-learning with ultrasound simulators [18].

Paramedics perceived ultrasound to be particularly useful in clinical scenarios they can actively influence, especially breathing problems, circulation problems, and vascular access. In contrast, no significant change was observed in perceived utility during cardiopulmonary resuscitation or polytrauma care. This likely reflects established algorithms emphasizing uninterrupted chest compressions during advanced life support (ALS) and rapid transport in trauma, as well as limited autonomous therapeutic options in these scenarios, which may have contributed to the low rate of echocardiography in advanced life support (ELS). Importantly, concerns that ultrasound might delay emergency operations were substantially reduced following training, with a median decline from 3 (IQR 3–4) before training to 2 (IQR 2–3) after training (*p* = 0.019), highlighting the value of hands-on simulation and time-management practice. Especially in FAST, it has already been demonstrated that training can reduce operating time by nearly 40% [19]. On the other hand, POCUS in ALS might also reduce hands-on time during resuscitation and reduce adherence to the ALS algorithms. Furthermore, a low diagnostic accuracy led to correct treatment decisions in only 44% [20]. Therefore, POCUS in an ALS setting requires specialized training, which should primarily focus on maintaining high-quality CPR while minimizing hands-off time during POCUS examinations and, secondarily, on interpreting relevant images during CPR.

A notable observation was the high proportion of referred patients requiring hospital admission (66 patients, 74.2%). This proportion exceeds the approximately 45% admission rate reported in comparable EMS cohorts [21]. Nevertheless, this comparison should be interpreted with caution. Our analysis did not include patients who did not go to the hospital, even though they may have required hospitalization, and therefore does not allow precise conclusions regarding the causal impact of ultrasound on admission decisions.

The observational data revealed good diagnostic accuracy for lung ultrasound and eFAST, while more complex examinations, such as focused echocardiography and RUSH, were used less frequently, likely due to technical and educational barriers. Here, we observed in personal debriefing conversations with the participants that there was a higher self-perceived burden in performing heart ultrasound than in other procedures. In contrast, Shadman et al. demonstrated that in the prehospital HEMS setting, echocardiography was the most common POCUS examination (60%), predominantly during cardiac arrest [4].

Sustaining ultrasound competence remains challenging due to low case frequency, emphasizing the need for ongoing training, structured governance, and supervision. Overall, the findings support integrating ultrasound into paramedic practice in the absence of a physician while highlighting the need for standardized curricula, quality assurance, and further studies evaluating patient-centered outcomes and system-level benefits.

### Limitations

Several limitations were identified. These limitations include small case numbers of 169 examinations compared to the total number of EMS responses. Additionally, the evaluated cohort may not fully represent the entire spectrum of diseases and disease severity. Consequently, diagnostic accuracy estimates may be overestimated and should be interpreted carefully. Additionally, final diagnoses were based on a composite reference standard including imaging and clinical documentation; differential verification bias cannot be fully excluded. Perceived impact on clinical and logistical decision-making was assessed by paramedic self-report and was not independently adjudicated, introducing potential reporting and observer bias.

## Conclusions

Paramedic-performed prehospital POCUS is feasible after structured training and can be integrated into routine prehospital care. In this exploratory real-world cohort, in some examinations, high diagnostic agreement with final in-hospital findings was observed.

However, given the exploratory design and its limitations, this study should be understood as an early feasibility and proof-of-concept project.

Further prospective studies with larger sample sizes, standardized reference diagnostics, and patient-centered outcome measures are required to robustly assess the diagnostic accuracy, reliability, and clinical impact of paramedic-performed POCUS in the prehospital setting.

## Supplementary Information

Supplementary Material 1.

## Data Availability

The datasets generated and analyzed during the current study contain prehospital clinical data and hospital-based diagnostic information that are subject to privacy and data protection regulations. Due to the nature of these data and applicable legal restrictions, they cannot be made publicly available. De-identified datasets may be shared upon reasonable request with the corresponding author and subject to a data use agreement and institutional approval.

## Abbreviations

AAA: Abdominal aortic aneurysm
ABCDE: Airway, Breathing, Circulation, Disability, Exposure
ALS: Advanced life support
CI: Confidence interval
CPR: Cardiopulmonary resuscitation
ED: Emergency department
eFAST: Extended focused assessment with sonography for trauma
ELS: Echocardiography in life support
EMS: Emergency medical services
FoCUS: Focused cardiac ultrasound
FOAM: Free open access medical education
HEMS: Helicopter emergency medical services
IV: Intravenous
LUS: Lung ultrasound
OR: Odds ratio
POCUS: Point-of-care ultrasound
PPV: Positive predictive value
NPV: Negative predictive value
RUSH: Rapid ultrasound in shock and hypotension
STROBE: Strengthening the Reporting of Observational Studies in Epidemiology
US: Ultrasound
USG-VA: Ultrasound-guided vascular access
