# Supplementary material for: Feasibility and diagnostic accuracy of paramedic-performed prehospital point-of-care ultrasound: a retrospective observational study
Source: Scand J Trauma Resusc Emerg Med. 2026 Mar 28;34:68. doi: 10.1186/s13049-026-01595-4 (PMC13063655; doi:10.1186/s13049-026-01595-4)
Supplement: Supplementary file 1 — Supplementary Material 1. [file 13049_2026_1595_MOESM1_ESM.docx]

**Supplementary Material**

**Prehospital POCUS Provider Questionnaire (Before and After Training)**

**1. Participant Characteristics**

- Years of professional experience in emergency medical services (EMS):

**2. Instructions**

Participants were asked to complete the questionnaire using a 5-point Likert scale:

1 = Strongly disagree

2 = Disagree

3 = Neither agree nor disagree

4 = Agree

5 = Strongly agree

**3. Prior Experience with Ultrasound**

Please indicate your level of agreement with the following statements:

- I have previously performed ultrasound examinations.
- I am familiar with ultrasound-assisted diagnostic procedures.
- Standardized ultrasound protocols are familiar to me.
- The Rapid Ultrasound in Shock and Hypotension (RUSH) protocol is familiar to me.
- The extended Focused Assessment with Sonography for Trauma (eFAST) protocol is familiar to me.
- Lung ultrasound is familiar to me.

**4. Expectations Regarding the Use of Prehospital Ultrasound**

Please indicate your level of agreement with the following statements:

- Ultrasound influences the selection of the destination hospital.
- Ultrasound is useful in patients with airway or breathing problems.
- Ultrasound is useful in patients with circulatory compromise.
- Ultrasound is useful during cardiopulmonary resuscitation.
- Ultrasound is useful in polytrauma patients.
- Ultrasound is useful for ultrasound-guided vascular access.
- Ultrasound improves prehospital treatment.
- Ultrasound delays prehospital treatment.

**5. Perceived Impact of Ultrasound Use in EMS *(posttraining only)***

Please indicate your level of agreement with the following statements:

- Ultrasound improves differentiation between possible diagnoses in the prehospital setting.
- Ultrasound influences therapeutic decision-making.
- Ultrasound influences transport-related decisions.
- Ultrasound helps to gain trust from patients and relatives.
- Ultrasound may increase my job satisfaction.

**6. General Attitudes Toward Ultrasound Diagnostics**

Please indicate your level of agreement with the following statements:

- Ultrasound is difficult to learn.
- Ultrasound should remain limited to hospital-based care.
- Ultrasound should remain reserved for physicians only.
- Ultrasound has no relevant role in prehospital emergency care.
- Ultrasound diagnostics are associated with excessive effort.

**7. Open Questions**

1. How many ultrasound examinations have you performed in total?
2. How many ultrasound examinations have you performed independently?
3. Which ultrasound applications have you personally performed?
4. Do you currently have little or no interest in ultrasound diagnostics?

**Prehospital POCUS Deployment Report Form**

**1. Ultrasound Protocol Applied**

☐ Lung ultrasound

☐ eFAST

☐ RUSH

☐ Cardiac arrest ultrasound

☐ Ultrasound-guided peripheral venous access

☐ Individual ultrasound examination (please specify):

**2. Impact on Tactical Decision Making**

As a result of ultrasound examination:

| **Item** | **Yes** | **No** |
| --- | --- | --- |
| Transport urgency increased | ☐ | ☐ |
| Transport urgency decreased | ☐ | ☐ |
| Emergency room prealert initiated | ☐ | ☐ |
| Emergency room prealert avoided | ☐ | ☐ |
| Physician backup requested | ☐ | ☐ |

*If “Yes”, please provide a brief comment.*

**3. Impact on Prehospital Patient Care**

As a result of ultrasound examination:

| **Item** | **Yes** | **No** |
| --- | --- | --- |
| Mission was unnecessarily delayed | ☐ | ☐ |
| Mission was meaningfully delayed | ☐ | ☐ |
| Differential diagnoses were excluded | ☐ | ☐ |
| Medication administration was indicated | ☐ | ☐ |
| Medication administration was avoided | ☐ | ☐ |
| Transport was indicated | ☐ | ☐ |
| Transport was avoided | ☐ | ☐ |

**4. Results and Handover**

As a result of ultrasound examination:

| **Item** | **Yes** | **No** |
| --- | --- | --- |
| Suspected diagnosis was confirmed | ☐ | ☐ |
| Patient condition improved | ☐ | ☐ |
| Acceptance by the receiving emergency department improved | ☐ | ☐ |
| Acceptance by hospital staff improved | ☐ | ☐ |
| Ultrasound images could be handed over | ☐ | ☐ |

- Destination hospital:
- Working diagnosis at handover:
- Feedback from the emergency department regarding ultrasound findings:
